# The CF-CIRC study: a French collaborative study to assess the accuracy of Cystic Fibrosis diagnosis in neonatal screening

**DOI:** 10.1186/1471-2431-6-25

**Published:** 2006-10-03

**Authors:** Isabelle Sermet-Gaudelus, Delphine Roussel, Stéphanie Bui, Eric Deneuville, Frédéric Huet, Philippe Reix, Gabriel Bellon, Gérard Lenoir, Aleksander Edelman

**Affiliations:** 1 Service de Pédiatrie Générale and CRCM, Hôpital Necker-Enfants Malades, 149 rue de Sèvres, 75743, Paris, France; 2 INSERM, U 806. Faculté de Médecine Necker, 156 rue de Vaugirard, 75730, Paris, France; 3 CRCM, Hôpital des Enfants. Place Amélie Raba Léon. 33076 Bordeaux, France; 4 CRCM, Hôpital Sud. 16 Boulevard de Bulgarie, BP 90327. 35203 Rennes, France; 5 CRCM, Hôpital d'Enfants du Bocage. 10 Bd du Maréchal de Lattre de Tassigny. 21034 Rennes, France; 6 CRCM, Hôpital Debrousse. 29 rue Sœurs Bouvier. 69322 Lyon, France; 7 Université Descartes Paris 5, Faculté de Médecine Necker, 156 rue de Vaugirard, 75730, Paris, France

## Abstract

**Background:**

Cystic fibrosis (CF) is caused by mutations in the gene encoding for the CF transmembrane conductance regulator (CFTR) protein, which acts as a chloride channel after activation by cyclic AMP (cAMP). Newborn screening programs for CF usually consist of an immunoreactive trypsinogen (IRT) assay, followed when IRT is elevated by testing for a panel of CF-causing mutations. Some children, however, may have persistent hypertrypsinogenemia, only one or no identified CFTR gene mutation, and sweat chloride concentrations close to normal values. *In vivo *demonstration of abnormal CFTR protein function would be an important diagnostic aid in this situation. Measurements of transepithelial nasal potential differences (NPD) in adults accurately characterize CFTR-related ion transport. The aim of the present study is to establish reference values for NPD measurements for healthy children and those with CF aged 3 months to 3 years, the age range of most difficult-to-diagnose patients with suspected CF. The ultimate goal of our study is to validate NPD testing as a diagnostic tool for children with borderline results in neonatal screening.

**Methods/Design:**

We adapted the standard NPD protocol for young children, designed a special catheter for them, used a slower perfusion rate, and shortened the protocol to include only measurement of basal PD, transepithelial sodium (Na^+^) transport in response to the Na^+ ^channel inhibitor amiloride, and CFTR-mediated chloride (Cl^-^) secretion in response to isoproterenol, a β-agonist in a Cl^- ^free solution.

The study will include 20 children with CF and 20 healthy control children. CF children will be included only if they carry 2 CF-causing mutations in the *CFTR *gene or have sweat chloride concentrations > 60 mEq/L or both. The healthy children will be recruited among the siblings of the CF patients, after verification that they do not carry the familial mutation.

**Discussion:**

A preliminary study of 3 adult control subjects and 4 children older than 12 years with CF verified that the new protocol was well tolerated and produced NPD measurements that did not differ significantly from those obtained with the standard protocol. This preliminary study will provide a basis for interpreting NPD measurements in patients with suspected CF after neonatal screening. Earlier definitive diagnosis should alleviate parental distress and allow earlier therapeutic intervention and genetic counseling.

## Background

Cystic Fibrosis (CF) is the most frequent life-limiting autosomal recessive disorder in the white [1]. It is caused by mutations in 2 alleles of the gene encoding for the CF transmembrane conductance regulator (CFTR) protein, which acts mainly as a cyclic AMP (cAMP)-activated chloride channel [2]. Classic CF is a multisystem disease characterized by elevated chloride concentrations in sweat, fat maldigestion due to pancreatic exocrine insufficiency, and chronic bacterial infection in the airways, which leads to terminal respiratory failure [3]. Newborn screening programs for CF (CFNBS) began in France 4 years ago because evidence shows that early diagnosis of this disease can prevent malnutrition and help to minimize lung damage [4]. Early diagnosis also allows parents to have earlier genetic counseling and to choose prenatal diagnosis in subsequent pregnancies, since only a minority of carrier couples can be directly detected. The French CFNBS is based on the immunoreactive trypsinogen (IRT) assay on dried blood, followed when IRT is elevated by analysis for a panel of CF-causing mutations [5]. IRT values tend to remain elevated for several months in babies with CF, because pancreatic trypsinogen leaks back through interstitial fluid due to partial obstruction of pancreatic ducts [6]. In some cases, however, IRT remains elevated even though CF cannot be formally diagnosed because only one mutation – or even none – is identified and the infant's sweat chloride concentration tends towards normal values [7]. These children may nonetheless develop symptoms consistent with CF or with a CF-related disorder [8]. The lack of specificity of IRT means, however, that they may also be false positives [9]. This presents a diagnostic dilemma. CF should be confirmed or ruled out as quickly as possible in these situations to alleviate parental distress and allow earlier therapeutic intervention and genetic counseling.

Standard diagnostic strategy calls for extensive analysis of the CFTR gene and repetition of the sweat Cl^- ^measurement [10,11]. Genetic studies, however, take several weeks and may find no useful information, neither confirming nor ruling out CF, such as when one or both mutant alleles remain unidentified or when the CF-causing nature of the mutations cannot be proven [12]. Sweat studies may also be unreliable, because not enough sweat is collected, or the values are borderline, or both [13].

**Figure 1:**
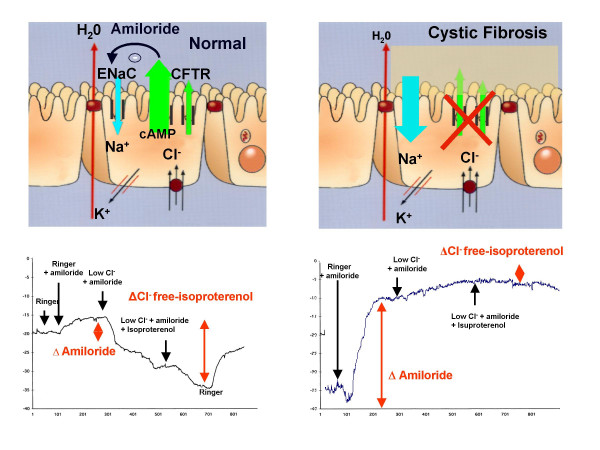
Ion transport in normal and CF nasal epithelium. Nasal PD measurements in a healthy subject and in a CF patient.

*In vivo *demonstration of abnormal CFTR-related ion transport across nasal epithelium could serve as an important diagnostic tool in these difficult situations. Sodium (Na^+^) transport and CFTR-related Cl^- ^transport can be measured electrically by recording the changes in the nasal transepithelial potential difference (PD) [14]. The profile of CF patients is characterized by hyperpolarization of basal PD, increased Na^+ ^channel activity, which can be detected by evaluating the magnitude of NPD recorded in the presence of a Na^+ ^channel blocker (amiloride), and defective adenosine cAMP-mediated Cl^-^ secretion, detected by the PD changes in response to β-adrenergic agonists in a low chloride solution (Figure 1). Nasal PD (NPD) measurement has been widely validated in adults [14] and provides a quick, easy, and painless tool to discriminate between adults with atypical CF and those presenting some CF symptoms without CF [15,16].

The NPD measurements in infants reported so far come mostly from case reports [17-19]. These few studies used either the equipment already validated in adults, or specially designed one-of-a-kind devices. It is important however to standardize the protocol and to verify that the reference data and patterns in infants are similar to the values previously validated in adults. We do not yet know, for example, whether airway epithelium undergoes maturation during the first months of life, as renal and sweat gland epithelia do [13]. Therefore, before this test is implemented as a diagnostic tool for cases with borderline observations in neonatal screening, there is an urgent need to obtain and validate reference data for NPD measurements in infants and very young children with CF and in healthy controls of the same age.

### Objectives

The aim of the study is to establish reference NPD values for both healthy children and those with CF aged 3 months to 3 years for measurements obtained with a simplified PD protocol and equipment specially designed for infants.

### Material

NPD Device and protocol development in infants and young children.

The protocol is derived from our previously published standardized protocol in older children [20].

PD between 2 silver/silver chloride electrodes is measured with a high input impedance (108–1012 ohm) low resistance voltmeter amplifier (Logan-Sinclair, Kent, UK), electrically isolated from the subject and linked to a computerized recording system. The skin of the inner forearm is slight abraded with a hand-held motor unit containing a diamond-tipped dental burr, to allow direct access to the subcutaneous space of the forearm and create a suitable site of zero potential. The reference electrode is taped over this site, and diluted (1:1) isotonic saline serum-electrode gel is injected through the hole into the electrode to allow electrical contact. The electrode is maintained in place by spars.

**Figure 2:**
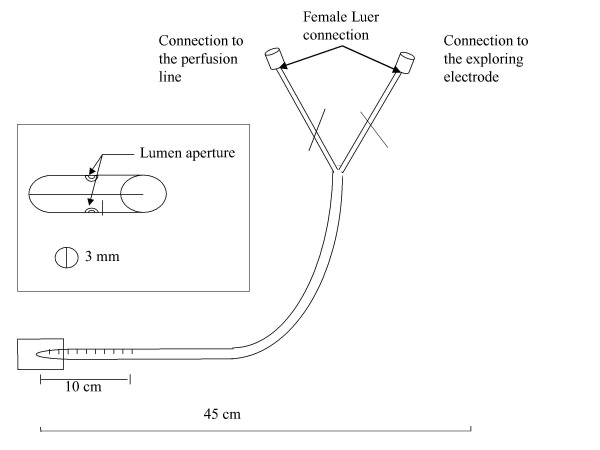
Pediatric catheter device.

The exploring electrode is connected to a nasal catheter filled with diluted ECG electrode gel. In collaboration with a manufacturer (Marquat Génie Médical, Boissy St   Léger, France, ) specialized in medical devices, we developed a manufactured single-use sterile catheter (Figure 2), This specially designed catheter carries the European Mark, granted by the appropriate body. The device is composed of a double lumen PVC tube, 45 cm in length, with an external diameter of 3 mm and graduated every 0.5 centimetre for about 10 cm from the distal extremity to control the position of fixation. One lumen (internal diameter: 1.0 mm) is filled with the 1:1 mixture of electrode gel and provides the bridge linked to the exploring electrode. The second lumen (internal diameter: 1.0 mm) allows the perfusion of different solutions by a pump that provides a continuous flow throughout the perfusion period.

For these measurements, children lie in a supine lateral recumbent position, either on the bed or in their mother's arms, with the head slightly elevated. Young children may require light sedation (intrarectal diazepam 0.5 mg/kg if younger than 6 months or midazolam 0.3 mg/kg for older infants). Conductivity of the bridge is assessed by measurements of skin palm PD at the beginning and end of the test, and acceptable values range from -30 mV upwards. Nasal PD is recorded in both nostrils as often as possible. Ringer's perfusion is begun at a rate of 1 ml/min and then increased according to tolerance to the optimal rate of 3 ml/min. Basal PD is recorded from the mucocutaneous junction at 2.5 mm intervals along the floor of the nose during perfusion of the Ringer's solution. The operator positions the probe at the point of maximal negative voltage and holds it in this position throughout the protocol.

After stabilization of basal PD, solutions are perfused in the following order: (1) 100 μM amiloride (sigma) in saline solution to block sodium current, (2) chloride-free solution containing 100 μM amiloride and 10 μM isoproterenol (Abbott) to drive chloride secretion and stimulate the cAMP-dependent CFTR-related Cl^- ^conductance. At the end of the perfusion of each solution, Ringer's solution is perfused for 30 seconds to ensure complete replacement of the liquid bathing the epithelium. Solutions are changed as soon as a steady voltage tracing is achieved for at least 45 seconds. The mean plateau voltage values for the last 30 seconds are taken as the final reading for each solution. For ease of reading, Δamiloride refers to the difference in NPD values between the plateau reached during perfusion with amiloride-containing Ringer's solution and that reached with amiloride in low chloride solution. Similarly ΔCl^-^free-isoproterenol refers to the net change between the amiloride-containing Ringer's solution and amiloride plus isoproterenol in low chloride solution. Both nostrils are explored as often as possible. The composition of the Ringer's solution is: NaCl: 8.18 g/l (140 mM); KCl: 0.44 g/l (6 mM); MgCl2 × 6H2O: 0.20 g/l (1 mM); Ca Cl2, 2H2O: 0.30 g/l (2 mM); glucose: 1.80 g/l (10 mM); and HEPES: 2.38 g/l (10 mM), water PPI qsp 1000 ml. Composition of the chloride-free solution is: Na Gluconate: 30.54 g/l (140 mM); K Gluconate: 1.44 g/l (6 mM); Mg C12 × 6H2O: 0.20 g/l (1 mM); Ca Cl2 × 2H2O: 0.30 g/l (2 mM); glucose: 1.80 g/l (10 mM); and HEPES: 2.38 g/l (10 mM)water PPI 1000 ml.

Pulse oxymetry and heart rate are monitored throughout the procedure. Weight is checked at 12 hours and 24 hours in children younger than 6 months. The procedure is stopped immediately if the child swallows the solution. Even if the child were to swallow 5 ml of the amiloride and isoproterenol, this would correspond to 0.5 mg amiloride and 0.01 mg of isoproterenol, a minute dose with no clinical consequences. Children who are sedated may not leave the hospital until completely awake.

Comparison of the standard method and the method modified for infants and young children

We compared the standard method and the method modified for infants in 3 healthy adults and 4 CF patients in a preliminary study. Each individual's tests were conducted at least 8 hours apart. The 3 healthy adult volunteers, mean age 33.3 (3.8) years, were asymptomatic for respiratory or gastrointestinal symptoms. They were nonsmokers with no history of nasal pathology. One healthy control took the modified test the day after the standard test and had rhinitis at that time. The mean age of the 4 CF patients was 15.2 (1.5) years. They all had chronic colonization with *Pseudomonas aeruginosa*. The same investigator performed all measurements.

## Methods/Design

### Patients and controls

This study will include children aged between 3 months and 3 years recruited from 9 French CF centers in the Paris area, Normandy, and southern France. Exclusion criteria for all participants will be upper respiratory tract infection during the 4 weeks before the study, passive smoking, atopy, nasal polyps, nasal surgery in the year before the measurement, and rhinitis or local nasal treatment during the month before the procedure.

The CF children must carry 2 CF-causing mutations in the CFTR and/or have a chloride sweat concentration > 60 mEq/L.

The healthy children will be recruited from the siblings of the CF patients. This strategy was chosen because we assume that very few healthy children younger than 3 years would normally undergo NPD testing for medical reasons. We think, however, that parents of children with CF may well agree to have their healthy child take this test to help advance research in this disease. Most often, the siblings of a CF child will have undergone genetic screening for the familial mutation either at antenatal diagnosis or after birth. If this is not the case, we will verify that their IRT level was normal at neonatal screening to decrease the risk of including heterozygotes and perform genetic testing to verify that they do not carry the familial mutation. These results will be communicated to the family before inclusion.

### Endpoints

All NPD tests will be performed by the same investigator. Endpoints for each nostril will be:

• mean basal PD value, that is, mean of the PD recordings from the mucocutaneous junction at 2.5 mm intervals along the floor of the nose

• lowest maximal basal PD as the maximal negative voltage

• Δamiloride as the differences in NPD values between the plateau reached during perfusion with amiloride in Ringer's solution and that reached with amiloride in low chloride solution

• ΔCl^-^free-isoproterenol as the net change between amiloride in Ringer's solution and amiloride plus isoproterenol in low chloride solution.

### Number of subjects

**Figure 3:**
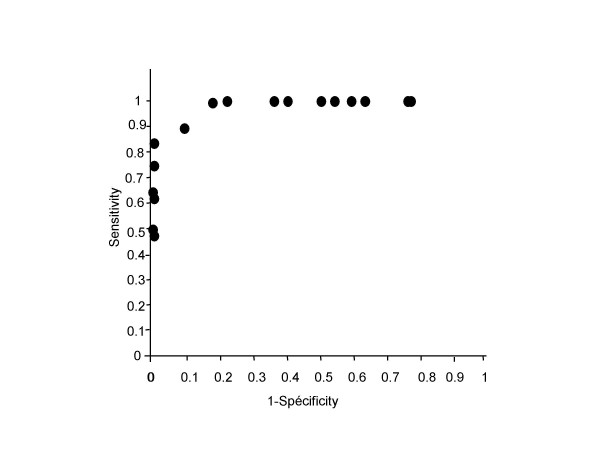
Sensitivity and specificity of ΔCl^-^free-isoproterenol for diagnosis of cystic fibrosis according to different threshold values.

Table 1 summarizes our reference data from patients aged 5–18 years. The indicator that best discriminates between patients with and without CF is ΔCl^-^free-isoproterenol (15). For these criteria, the reproducibility coefficient between both nostrils for the same patient is 89%, and measurement precision is 2.5 mV. The ROC curve shows the variation of the decision threshold (Figure 3). For ΔCl^-^free-isoproterenol = -8 mV, sensitivity is 0.90 and specificity 0.87, whereas sensitivity decreases to 0.8 at ΔCl^-^free-isoproterenol = -4 mV (specificity = 1), and to 0.7 for ΔCl^-^free-isoproterenol = -2 mV.

**Table 1:** Results of NPD measurements in CF patients, heterozygotes for 1 CF mutation and healthy controls.

Variables	CF (n = 37)	Htz (n = 37)	Ctrls (n = 29)	*p * CF *vs*. Htz	*p * CF *vs*. Ctrls	*p * Htz *vs*. Ctrls
Baseline NPD(mV)	-47(2.6)	-15.5(1.3)	-15(1.5)	<0.0001	<0.0001	0.9
Δamiloride (mV)	24(2.2)	6.2(0.9)	7.1(1.2)	<0.0001	<0.0001	0.69
Δchloride-free(mV)	0.5(1.1)	-6(0.8)	-10.1(1.5)	<0.0001	<0.0001	0.01
Δisoproterenol (mV)	-1(0.3)	-2.5(0.3)	-5(0.6)	0.004	<0.0001	0.003
ΔCl^-^free-Isoproterenol (mV)	1(1.3)	-8.2(0.9)	-15(1.7)	0.008	<0.0001	0.0002

Data are presented as mean (SEM)

Htz: heterozygotes; Ctrls: healthy controls

For Δ = 0.05 and a precision of 15%, the minimum number of subjects for each group is 20.

### Ethical considerations

The study was approved by the Necker-Enfants Malades Ethics Committee, and written informed consent will be obtained from the parents of each subject.

Because the aim of this study is to validate a diagnostic tool for cases considered borderline by neonatal screening for CF, that is, infants or children younger than 3 years, reference data for children younger than 3 years is essential. It is inappropriate to rely on values established in older children because of the possibility of airway epithelial maturation (given that we already know that sweat gland epithelium matures) (21). This study must therefore include children in the same age range as the children for whom this diagnostic tool may be used.

### Statistical analysis

Data will be presented as mean (SEM). Basal NPD, Δamiloride and Δchloride-free+isoproterenol, from the left nostril, the right nostril, and the average of both nostrils will be reported. Comparisons between groups will be analyzed with the Wilcoxon signed rank test for paired data and the Mann-Whitney test for unpaired data and will focus on the average values from both nostrils and on the nostril demonstrating the best change. A p-value less than or equal to 0.05 will be considered statistically significant.

## Discussion

Measurements of nasal PD in infants and young children present a variety of practical problems that this device and protocol overcome to the extent possible. Because infants cannot remain still, we shortened the procedure by adding isoproterenol directly in the low chloride perfusate after the perfusion of amiloride in Ringer's solution and by defining the steady plateau before perfusion change at 30 rather than 60 seconds, as in the standard method. The catheters used most frequently until now have been 8-Fr Foley pediatric urinary catheters modified by cutting the balloon area and placing it in the distal part or in single-lumen PE-50 tubing or umbilical vessel catheters (1–4). Southern and Knowles developed a single lumen polyethylene 10 tubing threaded over a 30-gauge needle acting both as electrode and perfusion line. This catheter is not for single use, however, and requires sterilization. This is an important limitation because of the risk of bacterial nasal carriage in CF and in view of international hygiene recommendations. To reduce the possibility of cross-contamination and minimize patient discomfort, we developed a single-use, sterile catheter specially designed for infant NPD in collaboration with a manufacturer specializing in biomaterials. The 3-mm diameter was designed for exploration of an infant's nostril. A catheter with a smaller diameter tends to slip out and could produce inaccurate measurements. The 1-mm diameter of the inner lumen allowed correct PD conductance after it was filled with diluted ECG gel. After extensive testing in our study, the catheter successfully underwent the procedure for European Mark approval.

The infant method differs from the standard method developed for adults in its use of a specially designed catheter, lower perfusion rate (1 ml/mn initially and 3 ml/mn versus 5 ml/mn at once) and shortened protocol that does not include the test of a low chloride solution. Volunteers and patients found both procedures equally tolerable. NPD values were consistent with previously published values in healthy subjects and CF patients [14,20].

There were no clinically significant differences between standard adult and pediatric methods for maximal basal PD and Δamiloride. Similarly, the response to isoproterenol in low chloride solution was not lower than with the adult method, thus indicating that the 2 methods provide comparable results under normal and disease conditions (Table 2).

**Table 2:** Comparison of the NPD results with the adult and the infant method

Method	Standard	Pediatric	Standard	Pediatric
Basal PD	-17.9(2.9)	-18.5(1.8)	- 58.9(3.2)	-59.3(1.7)
ΔAmiloride	7.1(1.3)	10.1(2.6)	29.7(6.3)	31.5(6.2)
ΔCl^-^free isoprotrenol	-26.3(6.9)	-23.3(8.4)	-0.2(0.2)	0(0)

## Conclusion

Neonatal screening for CF began in France 4 years ago. A substantial number of cases remain inconclusive because of intermediate sweat test values and no or only 1 identified mutation. The differential diagnosis between heterozygosity and CF is clearly crucial in these cases because some cases will develop atypical CF with fully expressed pulmonary involvement [8,21,22]. This study will provide the basis for interpreting findings from a new and rapid diagnostic tool in patients with suspected CF after neonatal screening. This should help clinicians to alleviate parental distress and provide earlier therapeutic interventions and genetic counselling [23].

## Competing interests

The author(s) declare that they have no competing interests.

## Authors' contributions

ISG conceived the study, carries out all the potential difference studies and wrote the manuscript and DR participated to the potential difference studies. SB, ED, FH, PD, GB, GL participated to the design of the study. AE participated in the design of the study and drafted the manuscript. All authors read and approved the final manuscript.

## Pre-publication history

The pre-publication history for this paper can be accessed here:


